# Development of ^52^Mn Labeled Trastuzumab for Extended Time Point PET Imaging of HER2

**DOI:** 10.1007/s11307-024-01948-4

**Published:** 2024-08-27

**Authors:** James M. Omweri, Shefali Saini, Hailey A. Houson, Volkan Tekin, Jennifer M. Pyles, Candace C. Parker, Suzanne E. Lapi

**Affiliations:** 1 Department of Chemistry, University of Alabama at Birmingham, Birmingham, AL 35205 USA; 2 Department of Radiology, University of Alabama at Birmingham, 1824 6th Ave S, WTI 310F, Birmingham, AL 35294 USA

**Keywords:** Manganese-52, Antibodies, Bifunctional chelators, Radiolabeling, Trastuzumab, Radiopharmaceuticals

## Abstract

**Purpose:**

Due to their long circulation time in the blood, monoclonal antibodies (mAbs) such as trastuzumab, are usually radiolabeled with long-lived positron emitters for the development of agents for Positron Emission Tomography (PET) imaging. Manganese-52 (^52^Mn, t_1/2_ = 5.6 d, β^+^  = 29.6%, E(β_ave_) = 242 keV) is suitable for imaging at longer time points providing a complementary technique to Zirconium-89 (^89^Zr, t_1/2_ = 3.3 d, β^+^  = 22.7%, E(β_ave_) = 396 keV)) because of its long half-life and low positron energy. To exploit these properties, we aimed to investigate suitable bifunctional chelators that could be readily conjugated to antibodies and labeled with ^52^Mn under mild conditions using trastuzumab as a proof-of-concept.

**Procedures:**

Trastuzumab was incubated with S-2-(4-isothiocyanatobenzyl)-1,4,7,10-tetraazacyclododecane tetraacetic acid (p-SCN-Bn-DOTA), 1-Oxa-4,7,10-tetraazacyclododecane-5-S-(4-isothiocyantobenzyl)-4,7,10-triacetic acid (p-SCN-Bn-Oxo-DO3A), and 3,6,9,15-tetraazabicyclo[9.3.1] pentadeca-1(15),11,13-triene-4-S-(4-isothiocyanatobenzyl)-3,6,9-triacetic acid (p-SCN-Bn-PCTA) at a tenfold molar excess. The immunoconjugates were purified, combined with [^52^Mn]MnCl_2_ at different ratios, and the labeling efficiency was assessed by iTLC. The immunoreactive fraction of the radiocomplex was determined through a Lindmo assay. Cell studies were conducted in HER2 + (BT474) and HER2- (MDA-MB-468) cell lines followed by *in vivo* studies.

**Results:**

Trastuzumab-Oxo-DO3A was labeled within 30 min at 37 °C with a radiochemical yield (RCY) of 90 ± 1.5% and with the highest specific activity of the chelators investigated of 16.64 MBq/nmol. The labeled compound was purified with a resulting radiochemical purity of > 98% and retained a 67 ± 1.2% immunoreactivity. DOTA and PCTA immunoconjugates resulted in < 50 ± 2.5% (RCY) with similar specific activity. Mouse serum stability studies of [^52^Mn]Mn-Oxo-DO3A-trastuzumab showed 95% intact complex for over 5 days. Cell uptake studies showed higher uptake in HER2 + (12.51 ± 0.83% /mg) cells compared to HER2- (0.85 ± 0.10%/mg) cells. PET images of mice bearing BT474 tumors showed high tumor uptake that was consistent with the biodistribution (42.02 ± 2.16%ID/g, 14 d) compared to MDA-MB-468 tumors (2.20 ± 0.80%ID/g, 14 d). Additionally, both models exhibited low bone uptake of < 1% ID/g.

**Conclusion:**

The bifunctional chelator p-SCN-Bn-Oxo-DO3A is promising for the development of ^52^Mn radiopharmaceuticals as it was easily conjugated, radiolabeled at mild conditions, and illustrated stability for a prolonged duration both *in vitro* and *in vivo*. High-quality PET/CT images of [^52^Mn]Mn-Oxo-DO3A-trastuzumab were obtained 14 d post-injection. This study illustrates the potential of [^52^Mn]Mn-Oxo-DO3A for the evaluation of antibodies using PET imaging.

**Supplementary Information:**

The online version contains supplementary material available at 10.1007/s11307-024-01948-4.

## Introduction

PET imaging of large constructs with long biological half-lives leverages the exquisite target specificity of monoclonal antibodies (mAbs) and other agents to assess target expression through tracer quantification in tumors, the targeting of novel drugs and in patient selection, stratification, and monitoring of treatment response [1–7]. Due to the long circulation time of mAbs in the blood, researchers have investigated imaging using long-lived radiometals such as ^64^Cu (t_1/2_ = 12.7 h), ^89^Zr (t_1/2_ = 3.3 d), ^86^Y (t_1/2_ = 14.7 h), ^111^In (t_1/2_ = 2.8 d) and ^52^Mn (t_1/2_ = 5.6 d) [8, 9].

Human epidermal growth factor receptor 2 (HER2) is overexpressed in 25–30% of breast cancers (BCa), often indicates an aggressive form of the disease, and is an important therapeutic target [10–17]. Trastuzumab is Food and Drug Administration (FDA) approved mAb that targets HER2 + BCa for treatment with efficacy depending on the HER2 expression levels [18].

^89^Zr is well-established positron emitter that has been used for HER2 + imaging [5, 9, 18–22]. Since the first evaluation of [^89^Zr]Zr-trastuzumab in humans by Dijkers et al. [22], several clinical trials using this radiotracer have been reported [23, 24]. [^89^Zr]Zr-trastuzumab PET/CT imaging of HER2 expression has been used to image metastases in patients with both IHC HER2 positive and negative BCa [22, 24, 25], to predict the efficacy of HER2-targeting antibody–drug-conjugates and support clinical decision making in treatment plans for BCa patients [21, 23].

^52^Mn is an emerging radiometal for PET imaging at late timepoints after injection due to its long half-life of 5.6 days, low average positron energy (242 keV), and adequate positron decay branching ratio (29.6%). These properties translate to PET images with favorable spatial resolution and motivate the use of ^52^Mn for imaging of long-lived biologics in addition to ^64^Cu (t_1/2_ = 12.7 h) and ^89^Zr (t_1/2_ = 3.3 d) [26–28]. A drawback to ^52^Mn is the emission of high energy photons (744 (90%), 936 (95%), and 1434 (100%) keV) which leads to additional dose to personnel and may hinder clinical translation [26, 29].

Recent studies have investigated ^52^Mn labeled mAbs for PET imaging [30]. Graves et al. evaluated [^52^Mn]Mn-DOTA-TRC105 in 4T1 tumor bearing mice, revealing tumor uptake of 19 ± 3%ID/g and some bone signal 120 h post injection [31]. Ferreira et al. developed YY146, a CD146-targeting mAb [32]. PET imaging of [^52^Mn]Mn-DOTA-YY146 in tumor bearing mice was used to assess CD146 expression levels in MDA-MB-435, MDA-MB-231 or MCF7 cell lines. MDA-MB-435 xenografts showed highest tumor uptake of 10.2 ± 0.5%ID/g and some bone signal at 120 h postinjection [32]. Csikos et al. synthesized and investigated the properties of [^52^Mn]Mn-DOTAGA-bevacizumab in KB-3–1 cervix carcinoma tumor-bearing mice through PET/MR imaging and observed high tumor uptake 10 days postinjection [33]. In a more recent study, Toan et al*.* developed a novel bispyclen-based BFC, BPPA, and used it to conjugate and radiolabel trastuzumab with ^52^Mn. They further evaluated the biological behavior of [^52^Mn]Mn-BPPA-trastuzumab in both HER2 + and HER2- mice models out to 10d following injection, using PET/MR imaging [34].

Our group has evaluated the chemistry of commercially available chelators which showed that Oxo-DO3A is a suitable chelator for ^52^Mn radiolabeling of biomolecules [35]. This work builds on these prior studies and investigates the suitability of three commercially available bifunctional chelators (BFCs) for conjugation with trastuzumab, radiolabeling, and long-term PET imaging of trastuzumab as a model antibody.

## Materials and Methods

All chemicals and other reagents were purchased from ThermoFisher Scientific (Hampton, NH) unless otherwise stated. Additional details are provided in the electronic supplementary material (ESM) section.

### Production and Quality Control of ^52^Mn

Production and purification of ^52^Mn followed previously published procedures [26, 29, 35, 36]. The apparent molar activity (AMA) of the resulting ^52^Mn was investigated through a chelation assay between ^52^Mn and serially diluted samples of the chelator DOTA following a published protocol [35].

### Conjugation of the BFCs with Trastuzumab

**Fig. 1:**
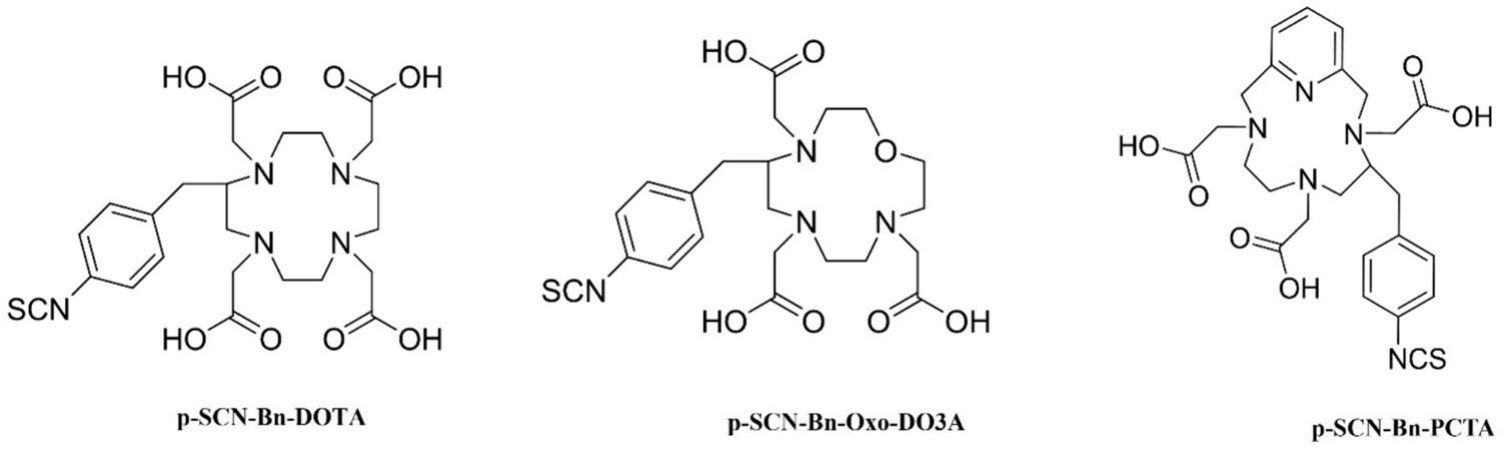
Selected chelators investigated for conjugation and radiolabeling of trastuzumab with ^52^Mn.

The BFCs: p-SCN-Bn-DOTA, p-SCN-Bn-Oxo-DO3A, and p-SCN-Bn-PCTA (Fig. 1) were conjugated to trastuzumab according to reported studies with some modifications [37–39]. Trastuzumab was reconstituted in water forming a 21 mg/mL stock solution and buffer exchanged into 0.1 M sodium bicarbonate buffer (pH 8.5) using 40 kDa zeba spin desalting columns. The BFCs were dissolved in 0.1 M sodium bicarbonate buffer and a tenfold molar excess of the chelators were incubated with trastuzumab for 1 h at 37 °C [38]. The resulting conjugates were purified, and buffer exchanged into 1 M HEPES (pH 7) using the 40 kDa desalting spin column.

A BCA assay was conducted to quantify the amount of trastuzumab after purification and the immunoconjugates were either stored at 4 °C or used immediately for radiolabeling.

### Radiolabeling of BFCs- Trastuzumab with ^52^Mn

10, 25, 50, and 100 μg of the immunoconjugates were combined with 2.8 MBq (75 μCi) of neutralized [^52^Mn]MnCl_2_ with 100 μL of buffer. DOTA-trastuzumab and PCTA-trastuzumab were radiolabeled in 0.1 M ammonium acetate, pH 4 while Oxo-DO3A-trastuzumab was radiolabeled in PBS, pH 7. The reaction mixtures were incubated at 37 °C for 1 h and the radiolabeling yield and purity was assessed by iTLC using a Si-60 iTLC paper developed in 50 mM DTPA (pH 7). Oxo-DO3A-trastuzumab was easily radiolabeled with the highest specific activity of the immunoconjugates and was chosen for further investigation.

### Serum Stability

To determine the stability of [^52^Mn]Mn-Oxo-DO3A-trastuzumab, 30 μL of the radiocomplex was added to 300 μL of mouse serum and incubated at 37 °C for 5 days. At pre-determined time points, 50 μL of serum/radiotracer mixture was combined with an equal volume of methanol to precipitate serum proteins and centrifuged at 3700 (revolutions per minute) RPM, 700 (relative centrifugal force) RCF 5 min). The supernatant was analyzed for % intact of the radiotracer using radio iTLC.

### *In vitro* Cell Studies

BT474 (HER2 +) and MDA-MB-468 (HER2-) cell lines were cultured in Gibbco’s high glucose Dulbecco’ Modified Eagle’s Medium (DMEM) supplemented with 10% fetal bovine serum (FBS) and 80 μM gentamicin. 1.8 mM of insulin was also added to the BT474 cell line media. The cells were maintained and grown in humidified incubators at 37 °C with 5% CO_2_ atmosphere.

#### Immunoreactivity

The immunoreactive fraction of [^52^Mn]Mn-Oxo-DO3A-trastuzumab was determined as described by Lindmo et al. [40]. Experimental details are provided in the ESM section.

#### Cell Binding

Cell binding assays were performed in BT474 (HER2 +) and MDA-MB-468 (HER2-) cell lines. 5 × 10^5^ cells were seeded in 6-well plates (n = 6) and incubated at 37 °C for 48 h before study. The incubating cell media was removed and 1 mL of fresh media containing 0.5 nM of [^52^Mn]Mn-Oxo-DO3A-trastuzumab was added to cells which were incubated at 37 °C for 2 h. After incubation, the radioactivity was removed, and the cells were washed in triplicate with ice-cold PBS. 500 μL of 0.2 M NaOH was added to lyse the cells before collection into microcentrifuge-tubes followed by a 500 μL wash of PBS. Associated radioactivity was measured using a Hidex gamma counter. To normalize the counts to the total protein amount, a BCA assay (ThermoFisher Scientific) was performed.

#### Internalization Assay

Internalization assays were carried out in BT474 (HER2 +) cells following a published procedure with slight modifications [41]. Additional experimental details can be found in the ESM section.

### Biodistribution and PET/CT Imaging of [^52^Mn]Mn-Oxo-DO3A-trastuzumab in Tumor Bearing Mice

All animal studies conducted in this work were performed using a protocol approved by the Institutional Animal Care and Use Committee (IACUC) at the University of Alabama at Birmingham and were compliant with national animal welfare policies and guidelines. The animals were allowed for one week to acclimate prior to any studies.

Mice meant for BT474 (HER2 +) tumors were implanted with a locally made 20 mg cholesterol pellet containing 0.72 mg of β-estradiol to hasten tumor growth as described by Ducharme et al. [15]. 8 × 10^6^ cells of either BT474 (HER2 +) or MDA-MB-468 (HER2-) in complete cell media were subcutaneously injected in the right shoulder. After 6 weeks, tumors were suitable for study (5 × 5 × 5 mm).

On the study day, [^52^Mn]Mn-Oxo-DO3A-trastuzumab was synthesized with a specific activity of 16.64 MBq/nmol (450 μCi/nmol). Approximately 0.08 ± 0.01 nmol, 1.29 ± 0.11 MBq (~ 35 ± 3 μCi) of the immunoconjugate was prepared in 100 μL. Mice (n = 4 per group) were anesthetized with 2.5% isoflurane in oxygen and were injected via the retroorbital sinus. At 3, 5, 7, 10, and 14 d post-injection time points, mice were imaged on a Sofie GNEXT PET/CT small animal scanner (Sofie Biosciences, Dulles, VA, USA). At each time point, 30 min of PET data were acquired immediately followed by a 3-min CT at 80 kVp for anatomical reference.

At 7 and 14 d post-injection time points, mice were euthanized, and organs were collected, weighed, and radioactivity measured. Radioactivity uptake was calculated as the percent injected dose per gram of tissue (% ID/g). Following reconstruction of the images, regions of interest (ROIs) covering the entire tumor area and corresponding to 3d (VOIs) volumes of interest were hand-drawn using CT images to determine the standardized uptake values (SUVs) using the VivoQuant imaging software.

### Statistical Analysis

Data were expressed as mean ± SD. Comparisons were made using GraphPad prism 9 software running student’s t-test and 2-way ANOVA utilizing Šídák's multiple comparisons test. P values of less than 0.05 were considered significant.

## Results

### Production and Quality Control of ^52^Mn

Approximately 205 ± 19 MBq (5.5 ± 0.5 mCi) ^52^Mn was produced at the end of a 4 h, 15 μA, and 12.5 MeV on target with an apparent molar activity of ~ 1080.1 ± 81.3 MBq/μmol. ^54^Mn (t_1/2_ = 312.1 d) was also observed constituting less than 0.4% of the total activity at the end of bombardment.

### Conjugation and Radiolabeling

**Fig. 2:**
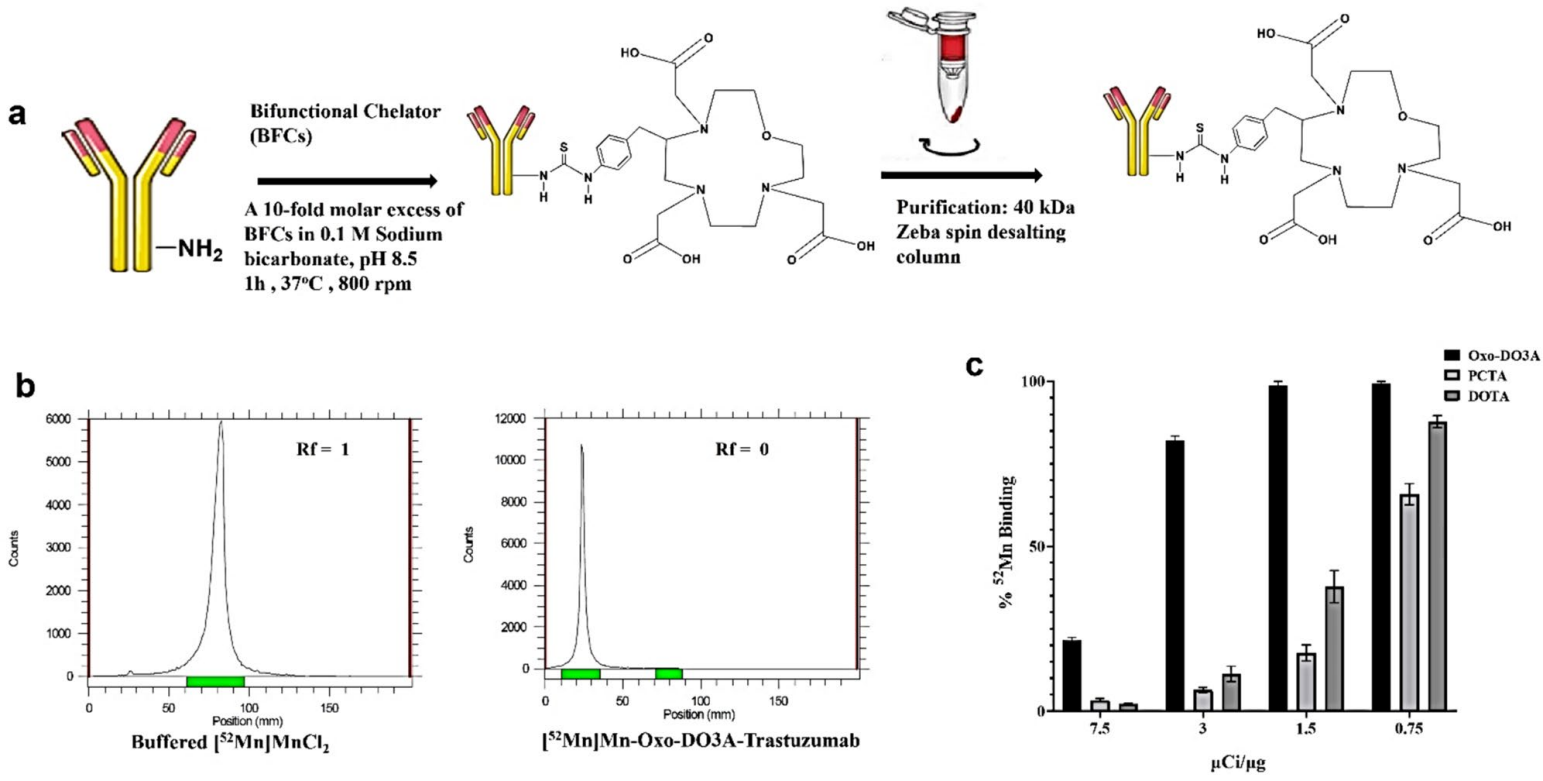
(**a**) Schematic diagram for steps involved in the conjugation of trastuzumab to different bifunctional chelators. (**b**) Radio TLC chromatograms for free ^52^Mn which moves with the solvent front and labeled trastuzumab which stays at the origin. (**c**) Comparison of percent labeling efficiency for chelators Oxo-DO3A, PCTA, and DOTA (*n* = *3*).

Trastuzumab was successfully conjugated to p-SCN-Bn-DOTA, p-SCN-Bn-Oxo-DO3A, and p-SCN-Bn-PCTA and radiolabeled with ^52^Mn. Oxo-DO3A-trastuzumab showed the highest RCY of 90 ± 1.5%. The radiolabeling efficiencies of DOTA and PCTA immunoconjugates with similar reaction ratios of 16.64 MBq/nmol all resulted in < 50 ± 2.5% RCY (Fig. 2c).

Oxo-DO3A-trastuzumab was readily radiolabeled with greater than 90% RCY and molar activity of 16.64 MBq/nmol (450 μCi/nmol). This radiotracer was purified resulting in > 98% purity and was chosen for further evaluation.

### Serum Stability and Immunoreactivity

**Fig. 3:**
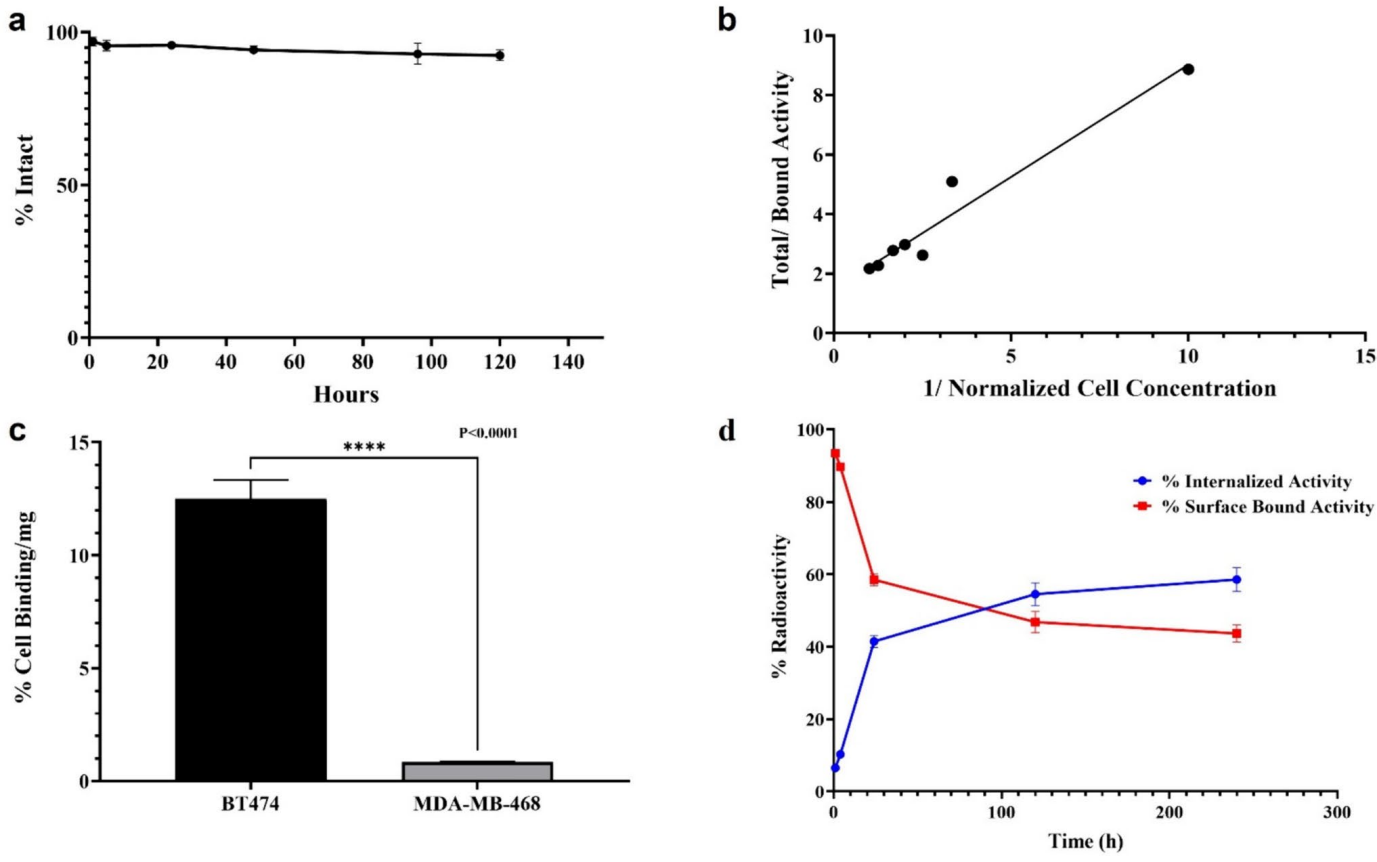
(**a**) Stability of [^52^Mn]Mn-Oxo-DO3A-trastuzumab in mouse serum (*n* = *3*), (**b**) immunoreactivity fraction of trastuzumab (**c**) Comparison of cell uptake of [^52^Mn]Mn-Oxo-DO3A-trastuzumab between BT474 and MDA-MB-468 cells (*n* = *6*) student unpaired t-test: ****P < 0.0001, and (**d**) cell internalization assay of [^52^Mn]Mn-Oxo-DO3A-trastuzumab in BT474 cells(*n* = *6*).

[^52^Mn]Mn-Oxo-DO3A-trastuzumab was stable in mouse serum with > 95% intact for more than 5 days (Fig. 3a) and immunoreactivity fraction was retained at 67 ± 1.2% (Fig. 3b).

### Cell Uptake and Internalization Assay

Cell binding illustrated significantly higher uptake in HER2 positive BT474 (12.51 ± 0.83% /mg) than in HER2 negative MDA-MB-468 (0.85 ± 0.10%/mg p < 0.0001) cells (Fig. 3c) after 2 h incubation at 37 °C. The internalized fraction was 10.25 ± 0.5% after 4 h incubation and 41.46 ± 1.68% after 24 h (Fig. 3d).

### PET Imaging and Biodistribution of [^52^Mn]Mn-Oxo-DO3A-trastuzumab

**Fig. 4:**
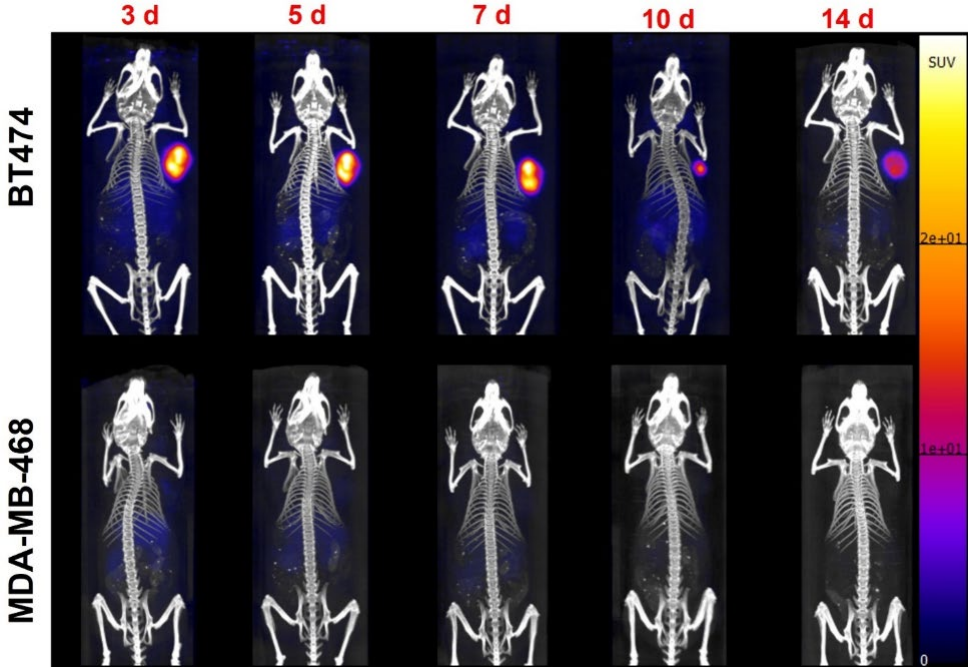
PET/CT images of [^52^Mn]Mn-Oxo-DO3A-trastuzumab in BT474 and MDA-MB-468 xenograft models showing images out to 14 d post injection.

**Fig. 5:**
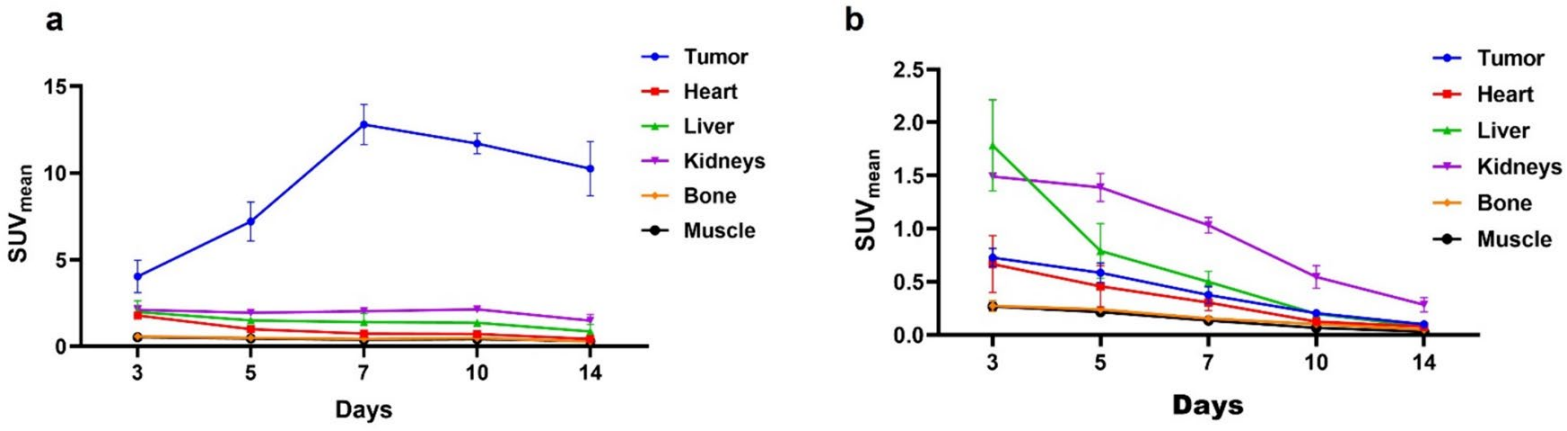
SUV_mean_ of selected tissues: **a**) HER2 + xenograft models and **b**) HER2- xenograft models.

PET images showed uptake and retention of the radiotracer in HER2 + tumors compared to the HER2- tumors (Fig. 4). Tumor uptake was highest at 7d post injection with SUV_mean_ values of 12.79 ± 1.16, *(n* = *4)* in HER2 + compared to 0.41 ± 0.08, *(n* = *4)* in HER2-; P < 0.0001. Gradual clearance of the radiotracer from the kidneys and liver was observed as shown in Tables 1 and 2. Metabolism is primarily hepatic as shown in the PET/CT images. The kinetics of blood pool clearance of the radiotracer is demonstrated by decrease in Heart SUVmean values determined at different time points as shown in Table 1 (HER2 +) and Table 2 (HER2-). Figure 5 shows clearance of the radiotracer in BT474 and MDA-MB-468 tumor models with high uptake and retention of the radiotracer 7 d after injection.

**Table 1:** SUV data of selected organs extracted from PET/CT images for BT474 (HER2 +) and xenograft models at different time points postinjection

Time (day)	Tumor		Heart		Liver		Kidney		Bone	
	HER2 +	HER2-	HER2 +	HER2-	HER2 +	HER2-	HER2 +	HER2-	HER2 +	HER2-
3	4.03 ± 0.94	0.73 ± 0.09	1.78 ± 0.17	0. 67 ± 0.26	1.20 ± 0.64	1.78 ± 0.43	2.11 ± 0.25	1.50 ± 0.03	0.58 ± 0.11	0.27 ± 0.05
5	7.20 ± 1.11	0.58 ± 0.09	0.98 ± 0.16	0.46 ± 0.20	1.50 ± 0.17	0.79 ± 0.30	1.94 ± 0.09	1.38 ± 0.14	0.48 ± 0.03	0.24 ± 0.03
7	12.79 ± 1.16	0.38 ± 0.08	0.76 ± 0.05	0.30 ± 0.08	1.40 ± 0.50	0.50 ± 0.10	2.02 ± 0.16	1.03 ± 0.07	0.42 ± 0.07	0.15 ± 0.02
10	9.20 ± 0.75	0.21 ± 0.03	0.70 ± 0.14	0.13 ± 0.02	1.35 ± 0.20	0.20 ± 0.01	2.13 ± 0.17	0.54 ± 0.11	0.47 ± 0.09	0.10 ± 0.02
14	10.24 ± 1.56	0.10 ± 0.01	0.41 ± 0.17	0.08 ± 0.02	0.85 ± 0.40	0.08 ± 0.01	1.48 ± 0.36	0.28 ± 0.06	0.28 ± 0.07	0.04 ± 0.01

**Table 2:** SUV data of selected organs extracted from PET/CT images for MDA-MB-468 (HER2-) and xenograft models at different time points postinjection

Time (day)	Tumor	Salivary glands	Heart	Lungs	Liver	Spleen	Pancreas	Kidneys	Muscle	Bone
3	0.76 ± 0.09	0.75 ± 0.20	0.67 ± 0.25	0.63 ± 0.27	1.95 ± 0.81	0.90 ± 0.26	1.15 ± 0.59	1.45 ± 0.13	0.26 ± 0.05	0.31 ± 0.05
5	0.57 ± 0.09	0.66 ± 0.14	0.45 ± 0.20	0.46 ± 0.17	1.08 ± 0.16	0.50 ± 0.18	0.64 ± 0.17	1.30 ± 0.07	0.21 ± 0.03	0.27 ± 0.04
7	0.41 ± 0.08	0.49 ± 0.12	0.29 ± 0.06	0.25 ± 0.09	0.76 ± 0.13	0.34 ± 0.13	0.56 ± 0.13	0.95 ± 0.06	0.13 ± 0.04	0.14 ± 0.05
10	0.20 ± 0.03	0.29 ± 0.10	0.13 ± 0.03	0.13 ± 0.03	0.26 ± 0.04	0.45 ± 0.22	0.23 ± 0.04	0.56 ± 0.10	0.07 ± 0.03	0.10 ± 0.03
14	0.10 ± 0.01	0.13 ± 0.04	0.08 ± 0.03	0.07 ± 0.03	0.11 ± 0.02	0.19 ± 0.06	0.10 ± 0.02	0.26 ± 0.08	0.03 ± 0.01	0.04 ± 0.01

**Fig. 6:**
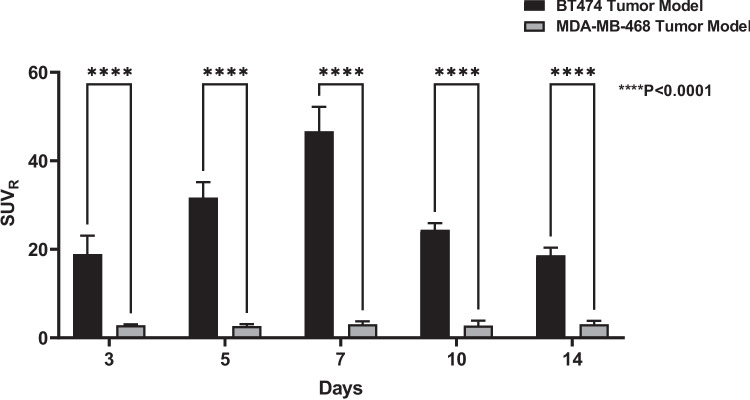
Tumor to muscle ratios in BT474 and MDA-MB-468 xenograft models at different time points postinjection. 2way ANOVA ****P < 0.0001.

The muscle tissue (along the thigh) was used as background activity. Tumor to muscle ratios using SUV_mean_ values for both HER2 + and HER2- tumors are shown in Fig. 6. The ratios for HER2 + tumors increased steadily and reached the peak at 7d postinjection.

**Fig. 7:**
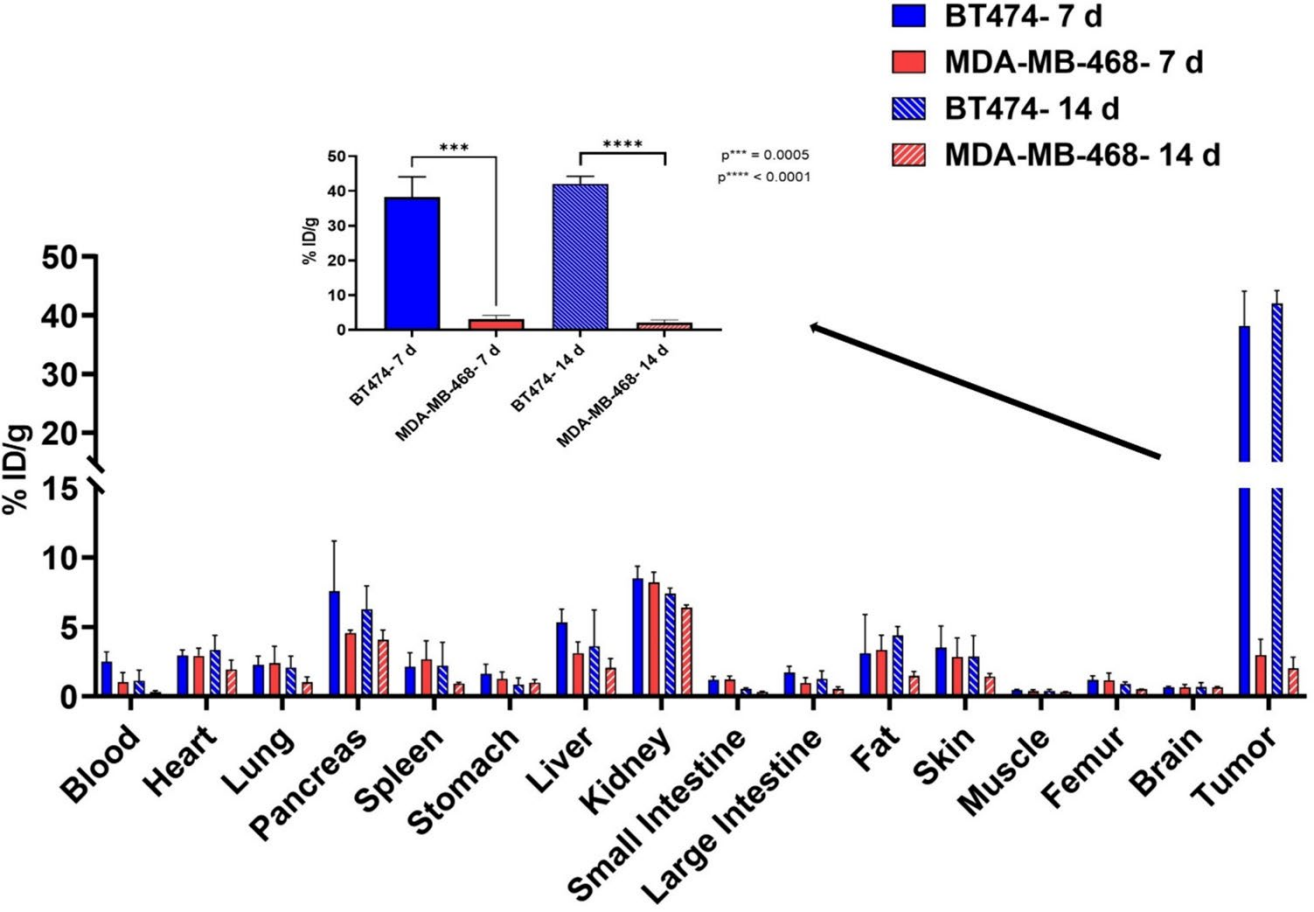
Biodistribution of [^52^Mn]Mn-Oxo-DO3A-trastuzumab in BT474 and MDA-MB-468 xenograft models. Student unpaired t-test, (n = 6) ****P < 0.0001.

The biodistribution of the radiotracer in mice bearing BT474 and MDA-MB-468 tumors showed similar distribution in all organs except for tumor uptake (Fig. 7). BT474 tumors showed high tumor uptake (38.19 ± 5.91%ID/g,7 d) compared to MDA-MB-468 tumors (2.98 ± 1.13%ID/g, 7 d p < 0.0001). The majority of trastuzumab was cleared from the blood by 14 d and notably, femur uptake was very low (0.90 ± 0.15%ID/g, 14 d) (Fig. 7). Table 3 gives a summary of the biodistribution of the radiotracer in both xenograft models.

**Table 3:** Biodistribution data (% ID/g) of [^52^Mn]Mn-Oxo-DO3A-trastuzumab in BT474 (HER2 +) and MDA-MB-468 (HER2-) xenograft models at 7 d and 14 d postinjection. (mean ± SD) n = 4

Tissue	7 d		14 d	
	HER2 +	HER2-	HER2 +	HER2-
Blood	2.50 ± 0.69	1.03 ± 0.70	1.13 ± 0.76	0.31 ± 0.12
Heart	2.93 ± 0.42	2.92 ± 0.57	3.36 ± 1.03	1.93 ± 0.70
Lung	2.29 ± 0.62	2.39 ± 1.22	2.09 ± 0.82	1.01 ± 0.41
Pancreas	7.58 ± 3.61	4.57 ± 0.21	6.28 ± 1.67	4.08 ± 0.68
Spleen	2.13 ± 1.01	2.68 ± 1.33	2.21 ± 1.68	0.93 ± 0.09
Stomach	1.63 ± 0.69	1.28 ± 0.48	0.87 ± 0.46	1.00 ± 0.22
Liver	5.30 ± 0.97	3.12 ± 0.81	3.61 ± 2.62	2.06 ± 0.67
Kidney	8.48 ± 0.89	8.21 ± 0.72	7.41 ± 0.39	6.39 ± 0.19
Small intestines	1.21 ± 0.24	1.22 ± 0.23	0.56 ± 0.06	0.33 ± 0.04
Large intestines	1.73 ± 0.45	0.95 ± 0.40	1.26 ± 0.59	0.56 ± 0.14
Fat	3.10 ± 2.78	3.36 ± 1.07	4.38 ± 0.64	1.48 ± 0.32
Skin	3.50 ± 1.58	2.86 ± 1.36	2.86 ± 1.52	1.43 ± 0.23
Muscle	0.47 ± 0.04	0.39 ± 0.09	0.40 ± 0.11	0.33 ± 0.01
Femur	1.18 ± 0.31	1.18 ± 0.51	0.90 ± 0.15	0.52 ± 0.02
Brain	0.67 ± 0.07	0.67 ± 0.19	0.68 ± 0.32	0.67 ± 0.06
Tumor	38.19 ± 5.91	2.98 ± 1.13	42.02 ± 2.16	2.20 ± 0.80

## Discussion

With increasing interest in antibody PET imaging, as well as the progress made in PET imaging techniques to enable the use of very low radiotracer concentrations including higher sensitivity scanners, more positron emitting radiometals are emerging as options alongside ^64^Cu, ^89^Zr, and ^86^Y [9]. For example, Berg et al. reported the feasibility of later time point PET imaging out to 30 d of rhesus monkeys after injection of ^89^Zr radiotracers using a long bore PET/CT [42].

For late time point imaging, ^89^Zr- chelates must remain stable to prevent release of ^89^Zr and subsequent bone uptake. Berg et al. examined the relationship between DFO linker moieties (DFO-Bz-NCS, DFO*-Bz-NCS, DFO-squaramide, and DFO*-squaramide) and stability and found that both DFO*-Bz-NCS and DFO*-squaramide resulted in a lower bone uptake [42]. However, while bone uptake has been reported in rodent models, this phenomenon has not been observed in clinical studies [43]. ^52^Mn is substantially longer lived than ^64^Cu, or ^86^Y and may be able to circumvent the enhanced bone uptake observed at long time points with some ^89^Zr agents [18, 44–46].

Intact antibodies have limitations of low blood clearance and non-specific uptake compared to low molecular weight targeting probes such as affibodies [15, 16]. Váradi et al. synthesized a bifunctional chelator 3,9-PC2ABn^*p*CO^_2_^H^, conjugated to a HER2 + targeting affibody and [[^52^Mn]Mn(3,9-PC2ABn^*p*MA^)(H2O)]Cys-HER2-affibody, was evaluated *in vivo*. They observed a tumor uptake of SUV_mean_: 0.63 in HER2 + tumors compared to our reported uptake of SUV_mean_: 4.03 3d postinjection [47].

The overexpression of HER2 in tumors is associated with aggressive disease, poor prognosis, and shorter overall survival [20]. The effectiveness of trastuzumab therapy depends on HER2 expression [10, 45, 48]. PET imaging with radiolabeled trastuzumab can select patients, monitor treatment response, and determine the optimal dose for a patient [18, 49].

Several preclinical studies and clinical trials involving [^89^Zr]Zr-trastuzumab have been reported in literature [10, 18, 21, 22, 25, 44, 49–52]. Due to  the long residence time in blood of trastuzumab, ^52^Mn with a longer physical half-life than ^89^Zr, can also be used to investigate trastuzumab and other antibodies at even later time points.

DOTA with formation constant of Log*K*_ML_(19.44) with Mn^2+^ compared to PCTA (16.88), and Oxo-DO3A(13.88) is among the most studied commercial chelator for ^52^Mn [53]. Biofunctionalized DOTA has been conjugated to different monoclonal antibodies for PET imaging with a significant bone uptake (> 5%ID/g) compared to < 1%ID/g reported herein. The authors attributed this bone uptake to a direct interaction between the bone and the DOTA-bound ^52^Mn [31–33]. In the current study, BFCs: p-SCN-Bn-DOTA, p-SCN-Bn-Oxo-DO3A, and p-SCN-Bn-PCTA were easily conjugated to trastuzumab following previously published procedures with slight modifications [37–39]. [^52^Mn]Mn-Oxo-DO3A-trastuzumab was obtained with the highest RCY of 90 ± 1.5% and a molar activity of 16.65 MBq/nmol (450 μCi/nmol) The radiotracer was purified further by a spin desalting column and > 98% purity obtained.

These results were comparable to those reported by Chang et al*.* who reported radiolabeling trastuzumab with ^89^Zr with a specific activity of 20.50 MBq/nmol (555 μCi/nmol) and a RCY of 78.4% [18]. Dijkers et al*.* reported the specific activities and labeling efficiencies of [^89^Zr]Zr-trastuzumab and [^111^In]In-trastuzumab as 10.07 MBq/nmol (270 μCi/nmol), 77.6% and 11.72 MBq/nmol (315 μCi/nmol), 89.3% respectively [51].

The radioimmunoconjugate demonstrated greater than 95% intact over a period of 5 days when incubated with mouse serum at 37 °C compared to less than 80% intact of [^52^Mn]Mn-BPPA-trastuzumab as reported by Toan et al. [34]. The average immunoreactive fraction was determined to be 67 ± 1.2% which was lower compared to > 80% reported with [^89^Zr]Zr-trastuzumab [18, 49, 51], but was within the acceptance criterion of 65% [49].

*In vitro* specificity of the radiotracer towards HER2 receptors illustrated a significantly higher % cell uptake in HER2 positive BT474 (12.51 ± 0.83%/mg) than in HER2 negative MDA-MB-468 (0.85 ± 0.10%/mg). Cell binding assays confirmed the HER2 status, however there are reports in the literature that HER2 status can change over time which is a limitation of the study [54].

An internalization assay of [^52^Mn]Mn-Oxo-DO3A-trastuzumab using the HER2 + BT474 cells demonstrated that 10.26 ± 0.5% of the total activity was internalized after 4 h which later increased to 41.46 ± 1.68% after 24 h incubation at 37 °C. Similar results have been reported with [^111^In]In-trastuzumab and [^89^Zr]Zr-trastuzumab utilizing the internalizing properties of trastuzumab antibody and the residualizing nature of ^111^In and ^89^Zr radionuclides [9, 37].

*In vivo* targeting and biodistribution of [^52^Mn]Mn-Oxo-DO3A-trastuzumab was investigated in nude mice bearing HER2 + BT474 and HER2- MDA-MB-468 tumors. PET images obtained at different time points postinjection: 3, 5, 7, 10, and 14 d showed excellent uptake and retention of the radiotracer with clearance of activity from the liver and the kidney. We hypothesize that the Mn complex stays in Mn^2+^state but this has not been definitively shown to date.

[^52^Mn]Mn-Oxo-DO3A-trastuzumab images showed high tumor uptake out to 14 d post injection. Except for tumor uptake, the biodistribution of the [^52^Mn]Mn-Oxo-DO3A-trastuzumab in both HER2 + and HER2- tumor bearing mice was similar. BT474 tumors showed high tumor uptake (42.02 ± 2.16%ID/g, 14 d) compared to MDA-MB-468 tumors (2.20 ± 0.80%ID/g, 14 d). Literature reports similar biodistribution profiles of [^111^In]In-trastuzumab and [^89^Zr]Zr-trastuzumab in mice [18, 37, 50–52]. Chang et al*.* reported 28.83 ± 1.33% ID/g in HER2 + tumors and 7.91 ± 0.96%ID/g in HER2- tumors 4 d after injection of [^89^Zr]Zr-trastuzumab. Dijkers et al. reported an uptake of [^89^Zr]Zr-trastuzumab in HER2 + tumors of 33.4 ± 7.6%ID/g and 7.1 ± 0.7%ID/g in HER2- tumors 6 d postinjection compared to the uptake reported in this study of 34.9 ± 3.0%ID/g in HER2 + and 3.0 ± 1.0%ID/g in HER2- 7 d postinjection [51]*.* Recently, Toan et al., developed a novel bispyclen-based BFC, BPPA, which was used to radiolabel trastuzumab with ^52^Mn with a specific activity of 0.085 MBq/μg compared to 0.111 MBq/μg reported herein. *In vivo* investigation of this radiotracer using PET/MR demonstrated high tumor uptake with persistent accumulation of the radiotracer in the liver, kidney, and pancreas where free ^52^Mn is known to accumulate [34]. [^52^Mn]Mn-Oxo-DO3A-trastuzumab demonstrated a slightly higher tumor uptake (SUV_mean_ 12.79 ± 1.16, 7d) compared to [^52^Mn]Mn-BPPA-trastuzumab (SUV_mean_ 10.08 ± 2.18) reported by Toan et al. [34].

Uptake of ^89^Zr in bone has been associated with the instability of some ^89^Zr complexes used in PET imaging. Free ^52^Mn is known to accumulate in the salivary glands, kidneys, liver, pancreas, and spleen but does not localize in the bone [55]. Biologically, free Mn^2+^ has been shown to exhibit similar behavior to calcium. Observations have been made regarding the diffusion of Mn^2+^ through the voltage dependent calcium channels (VDCCs) and its subsequent accumulation in the pancreas. This feature has been used to track neuronal pathways and monitor β-cell mass as type 1 diabetes progresses [34, 56].

In this study, bone uptake with [^52^Mn]Mn-Oxo-DO3A-trastuzumab was less than 1%ID/g 7 d post injection compared to > 5%ID/g when [^89^Zr]Zr-DFO-trastuzumab is used for late time point postinjection [18, 25, 44–46, 57, 58].

## Conclusion

In this work, we have demonstrated that the bifunctional chelator Oxo-DO3A is a suitable ^52^Mn chelator which is readily conjugated, radiolabeled at mild conditions, and illustrated stability over a prolonged duration *in vitro* and *in vivo.* [^52^Mn]Mn-Oxo-DO3A-trastuzumab was synthesized with > 98% purity and was stable in mouse serum for over 5 days and demonstrated high specificity towards the HER2 receptors through cell binding and internalization assays.

PET imaging of [^52^Mn]Mn-Oxo-DO3A-trastuzumab in a xenograft model showed high tumor to muscle ratio up to 14 d postinjection in HER2 + tumors. We also observed a low bone uptake (< 1% ID/g) with the ^52^Mn radiotracer compared to ^89^Zr labeled trastuzumab (> 5% ID/g). Finally, high uptake of [^52^Mn]Mn-Oxo-DO3A-trastuzumab in HER2 + ( 38.2 ± 5.9%ID/g 7d) similar to that of [^89^Zr]Zr-trastuzumab in HER2 + tumors ( 33.4 ± 7.6%ID/g 6 d postinjection) can allow the use of ^52^Mn in a complementary fashion to ^89^Zr especially in late time point antibody PET imaging to study pharmacokinetics of new antibodies and antibody drug conjugates.

## Supplementary Information

Below is the link to the electronic supplementary material.

Supplementary file1 (DOCX 25 KB)

## Data Availability

The authors declare that the data supporting the findings of this study are available within the paper. Should any new data files be needed in another format they are available from the corresponding author upon reasonable request.
