# Supplementary material for: Development of 52Mn Labeled Trastuzumab for Extended Time Point PET Imaging of HER2
Source: Mol Imaging Biol. 2024 Aug 27;26(5):858–68. doi: 10.1007/s11307-024-01948-4 (PMC11436409; doi:10.1007/s11307-024-01948-4)
Supplement: Supplementary file 1 — Supplementary file1 (DOCX 25 KB) [file 11307_2024_1948_MOESM1_ESM.docx]

**Electronic supplementary Materials**

**Materials and methods**

Natural chromium powder (5N purity), tantalum sheets (3N8 purity), and aluminum sheets (5N purity) were all purchased from ESPI Metals (Ashland, OR). Viton O-rings were obtained from McMaster-Carr (Elmhurst, IL). The 10 mm diameter ID dry pressing die set was obtained from Across International (Livingston, NJ). 1 mL SPE tubes with frits were obtained from Millipore Sigma (Burlington, MA). AG1-X8 resin analytical grade 200–400 mesh chloride form was obtained from Bio-Rad (Hercules, CA). S-2-(4-Isothiocyanatobenzyl)-1,4,7,10-tetraazacyclododecane tetraacetic acid (p-SCN-Bn-DOTA), 1-Oxa-4,7,10-tetraazacyclododecane-5-S-(4-isothiocyantobenzyl)-4,7,10-triacetic acid (p-SCN-Bn-Oxo-DO3A), and 3,6,9,15-Tetraazabicyclo[9.3.1] pentadeca-1(15),11,13-triene-4-S-(4-isothiocyanatobenzyl)-3,6,9-triacetic acid ( p-SCN-Bn-PCTA) bifunctional chelators (BFCs) were all obtained from Macrocyclics Inc. (Plano, TX). Zeba™ Spin Desalting Columns, 40K MWCO, 0.5 mL (ThermoFisher Scientific). BT474 (HER2+) and MDA-MB-468 (HER2-) cells were all purchased from American Type Culture Collection (Manassas, VA). Trastuzumab (Herceptin™) was purchased from Genentech, South San Francisco, CA). Mouse serum was sourced from EMD Millipore Corporation (Temecula, CA). Five-week-old female athymic nude mice were obtained from Charles River Laboratories (Wilmington, MA). iTLC Si-60 paper was obtained from Sorbtech Technologies (Norcross, GA).

**Immunoreactivity**

Briefly, seven different serial dilutions of HER2+ BT474 cells: 2.5 x 10^6^ , 2.0 x 10^6^ , 1.5 x 10^6^ , 1.25 x 10^6^ , 1.0 x 10^6^ , 0.75 x 10^6^ , and 0.25 x 10^6^ cells in a total volume of 500 μL were prepared in PBS supplemented with 1% BSA. Approximately 5.56 x 10^-4^ MBq (3.34 x 10^-5^ nmol) of [^52^Mn]Mn-Oxo-DO3A-Trastuzumab was added to each of the tubes (n=3) and incubated for 1 h at room temperature. Tubes were centrifuged for 2 min at 600 x g and the supernatant was discarded. The cell pellets were resuspended and washed twice using ice-cold PBS. Finally, radioactivity associated with the cell pellet was counted on a HIDEX AGM gamma counter (Turku, Finland). This data was used to perform a linear regression analysis on a plot of (total/bound) activity against normalized cell concentration and the immunoreactive fraction was determined from the inverse of the y-intercept.

**Internalization assay**

Briefly, 5× 10^5^ cells were seeded in 12-well plates and incubated overnight at 37 °C. 1 mL of fresh media containing 0.5 nM of [^52^Mn]Mn-Oxo-DO3A-Trastuzumab was added to the wells after removing the culture media and the plates were incubated at 37 °C for 1, 4, 24, 120, and 240 h. At each time point, the media was removed, and cells were washed 2 times with ice-cold PBS. To determine the surface-bound fraction, 0.5 mL of 0.1 M citric acid was added to each well and incubated for 5 min. The fraction was collected into an Eppendorf tube followed by the addition of 0.5 mL gentle wash of PBS. To determine the internalized activity, 0.5 mL of 0.2 M NaOH was added to each well and incubated at 37 °C for 5 min. This fraction was collected in a separate tube followed by the addition of 0.5 mL of PBS wash. The results were expressed as a percentage of the total activity that was present in each of the two fractions.
